# Effect of histology on the efficacy of immune checkpoint inhibitors in advanced non-small cell lung cancer: A systematic review and meta-analysis

**DOI:** 10.3389/fonc.2022.968517

**Published:** 2022-11-10

**Authors:** Feng Li, Suokai Zhai, Zhuoheng Lv, Ligong Yuan, Shuaibo Wang, Donghui Jin, Hang Yi, Li Fu, Yousheng Mao

**Affiliations:** ^1^ Department of Thoracic Surgery, National Cancer Center/National Clinical Research Center for Cancer/Cancer Hospital, Chinese Academy of Medical Sciences and Peking Union Medical College, Beijing, China; ^2^ Department of Cardiothoracic Surgery, Zibo First Hospital, Weifang Medical University, Zibo, Shandong, China

**Keywords:** non-small-cell lung cancer, histology, immune checkpoint inhibitors, efficacy, meta-analysis

## Abstract

**Background:**

Little is known about the effect of histology on the efficacy of immune checkpoint inhibitors (ICI) in non-small-cell lung cancer (NSCLC). We conducted a systematic review and meta-analysis to assess the potential differences in the efficacy of ICIs between squamous NSCLC (SQ-NSCLC) and non-squamous NSCLC (non-SQ-NSCLC).

**Methods:**

Systematic searches of PubMed, Embase, Scopus, and Cochrane Library databases were conducted. All randomized clinical trials of ICIs with available hazard ratios (HR) for progression-free survival (PFS) or overall survival (OS) according to histology were included. The primary endpoint was to assess the difference in the efficacy of ICIs between SQ-NSCLC and non-SQ-NSCLC, measured by the ratio of the HR in SQ-NSCLC to the HR in non-SQ-NSCLC (RHR).

**Results:**

A total of 40 trials were included in the meta-analysis. ICI monotherapy could improve OS in both SQ-NSCLC (OS-HR 0.71, 95% CI 0.65-0.77) and non-SQ-NSCLC (OS-HR 0.80, 95% CI 0.73-0.87) while OS benefit was larger in SQ-NSCLC (OS-RHR 0.89, 95% CI 0.80-0.99). In terms of PFS, ICI monotherapy could reduce the risk of progression by 35% (PFS-HR 0.65, 95% CI 0.56-0.77) in SQ-NSCLC while the PFS benefit was smaller (10%) and not statistically significant in non-SQ-NSCLC (PFS-HR 0.90, 95% CI 0.76-1.07). Similarly, ICI-based combination treatments could reduce the risk of both progression and death in SQ-NSCLC (OS-HR 0.70, 95% CI 0.61-0.80; PFS-HR 0.56, 95% CI 0.48-0.65) and non-SQ-NSCLC (OS-HR 0.78, 95% CI 0.74-0.83; PFS-HR 0.63, 95% CI 0.57-0.69) while the survival benefits were larger in SQ-NSCLC (OS-RHR 0.83, 95% CI 0.70-0.99; PFS-RHR 0.82, 95% CI 0.70-0.96).

**Conclusions:**

ICIs could deliver survival benefits in both SQ-NSCLC and non-SQ-NSCLC while the magnitude of survival benefits was histology-dependent. Future researches should consider the effect of histology on the efficacy of ICIs.

**Systematic Review Registration:**

https://www.crd.york.ac.uk/prospero/, identifier [CRD42022299603].

## Introduction

Lung cancer remains the leading cause of cancer death worldwide (1). Approximately 80–90% of lung cancers are non-small-cell lung cancer (NSCLC), which is typically classified into two major types according to histology: squamous NSCLC (SQ-NSCLC) and non-squamous NSCLC (non-SQ-NSCLC). Compared with non-SQ-NSCLC, SQ-NSCLC has fewer treatment options due to absence of targetable mutations (2). Platinum-based chemotherapy (CT) used to be the standard treatment for advanced SQ-NSCLC; however, it has limited survival benefits (3). The introduction of immune checkpoint inhibitors (ICI) has revolutionized the therapeutic landscape of patients with NSCLC (4). ICIs are associated with improved overall survival (OS), lower toxicity, and better quality of life compared with CT and have emerged as the new mainstay of treatment for both SQ-NSCLC and non-SQ-NSCLC (5–8).

Despite the higher efficacy of ICIs compared with CT, only a subset of patients responded to this class of therapy. Previous studies have shown that the beneficiary population of ICI monotherapy is limited to those with high programmed death ligand 1 (PD-L1) expression or high tumour mutational burden (TMB) (7–11). Since ICI monotherapy is only suitable for a specific subset of patients, studies have focused on ICI-based combination treatments to further improve the benefit. ICI-based combination therapies include ICIs plus CT (12–15), dual ICIs (16) and dual ICIs plus CT (17). The combination of ICI and CT is able to synergistically promote the respective anticancer efficacy of these two classes of drugs (18, 19). Dual ICIs targeting both programmed cell death 1(PD-1)/PD-L1 and cytotoxic T-cell lymphocyte antigen-4 (CTLA-4), with complementary mechanisms of action, would restore the function of existing anti-tumour T cells and induce T-cell proliferation and *de-novo* anti-tumour T-cell responses (20, 21). When tested in randomized clinical trials (RCT), ICI-based combination treatments consistently improved the clinical outcomes of both SQ-NSCLC and non-SQ-NSCLC (12, 14, 16, 17).

Nevertheless, SQ-NSCLC and non-SQ-NSCLC are two distinct diseases with different origins (22). Beyond the morphologic difference, tumour microenvironment and mutational profiles differ markedly in these two histological subtypes (23, 24). It is plausible that these differences may influence the response to therapeutic agents, such as ICIs, and subsequent prognosis. However, whether histology could influence the efficacy of ICIs in NSCLC has not been fully evaluated yet. Therefore, we conducted a comprehensive systematic review and meta-analysis to uncover the potential differences in the clinical efficacy of ICIs between SQ-NSCLC and non-SQ-NSCLC.

## Methods

### Protocol and registration

This systematic review and meta-analysis was carried out following the Preferred Reporting Items for Systematic Reviews and Meta-analysis (PRISMA) guidelines (25), and the study protocol was registered in PROSPERO (CRD42022299603).

### Search strategy and study selection

A comprehensive literature search in PubMed, Embase, Scopus, and Cochrane Library databases (from inception to October 16, 2021) was performed by two authors independently to identify RCTs that compared ICIs with CT in NSCLC. We also reviewed the abstracts and presentations from major conferences including American Society of Clinical Oncology, European Society for Medical Oncology, and World Conference on Lung Cancer. The following terms were used: “non-small cell lung cancer”, “carcinoma, non-small-cell lung”, “immune checkpoint”, “immunotherapy”, “programmed death”, “programmed cell death”, “PD-1”, “PD-L1”, “cytotoxic T lymphocyte-associated protein-4” and “CTLA-4”. We also checked the reference lists of all relevant studies for additional suitable studies.

Studies were considered eligible if they met the following criteria: (1) RCTs; (2) compared ICIs (as monotherapy or in combination with other agents) with CT; (3) had data available on hazard ratios (HR) for progression-free survival (PFS) or OS according to histological type; (4) published in English. The following studies were excluded: (1) studies with treatment regimens other than ICIs and CT, such as radiotherapy and surgery; (2) studies without sufficient data; (3) reviews, case reports, guidelines, and letters. If duplicate articles were identified, only the most recent article with complete data was selected.

Two authors independently reviewed the retrieved articles to decide which study to retain, and discrepancies were discussed and resolved with the consensus of all authors.

### Data extraction and quality assessment

For each study, two investigators independently extracted the following information: trial name, year of publication, study phase, treatment regimens, line of therapy, number of patients, histological subtypes, class of ICI, HRs for PFS (PFS-HR), HRs for OS (OS-HR) and their 95% confidence intervals (CI). When HRs and/or the corresponding CIs were not numerically reported in the article, we extracted and calculated by plotting on a forest plot with a logarithmic scale. We assessed the methodological quality of studies using the Jadad scoring system, which assessed the quality of double-blinding and randomization as well as the flow of patients (26). This scoring system provides a Jadad score between 0 (poor methodological quality) and 5 (high methodological quality).

### Statistical analysis

Two meta-analyses were conducted. The first meta-analysis focused on the survival benefits of ICIs compared with chemotherapy according to histological subtypes, measured by the pooled HRs in all included studies. The second meta-analysis focused on the differences in the efficacy of ICIs between SQ-NSCLC and non-SQ-NSCLC, measured by the pooled ratios of HRs (RHR) in studies with paired subgroups (SQ-NSCLC vs non-SQ-NSCLC).

The primary endpoint was the ratio of the HR in SQ-NSCLC to the HR in non-SQ-NSCLC (RHR), which showed the difference in the efficacy of ICIs between SQ-NSCLC and non-SQ-NSCLC. As described previously (27, 28), we first calculated a trial-specific RHR in each study with paired subgroups and then these trial-specific RHRs were combined across trials to obtain a pooled RHR and a P-value for interaction (P_interaction_). A pooled RHR lower than 1 indicated greater efficacy in SQ-NSCLC, and higher than 1 indicated greater efficacy in non-SQ-NSCLC.

Heterogeneity among studies was assessed using Q test and I (2) test with significance set at P < 0.05 or I (2) >50%. A random-effects model was used when there was substantial heterogeneity. Publication bias was assessed using the Deeks funnel plot with Egger’s test (29). To assess the stability of the results, predefined subgroup analyses stratified by the type of immunotherapeutic strategy (ICI monotherapy or ICI-based combination treatments) and the class of ICI (anti-PD-1, anti-PD-L1, or anti-CTLA-4) were performed. Sensitivity analyses were conducted by removing studies that enrolled patients with epidermal growth factor receptor (*EGFR*) or anaplastic lymphoma kinase (*ALK*) mutations. All tests were two-sided and a P-value < 0.05 was considered statistically significant. All analyses were performed with STATA 16.0 (Stata Corp, College Station, Texas).

## Results

### Search results and characteristics of the included studies

**Figure 1:**
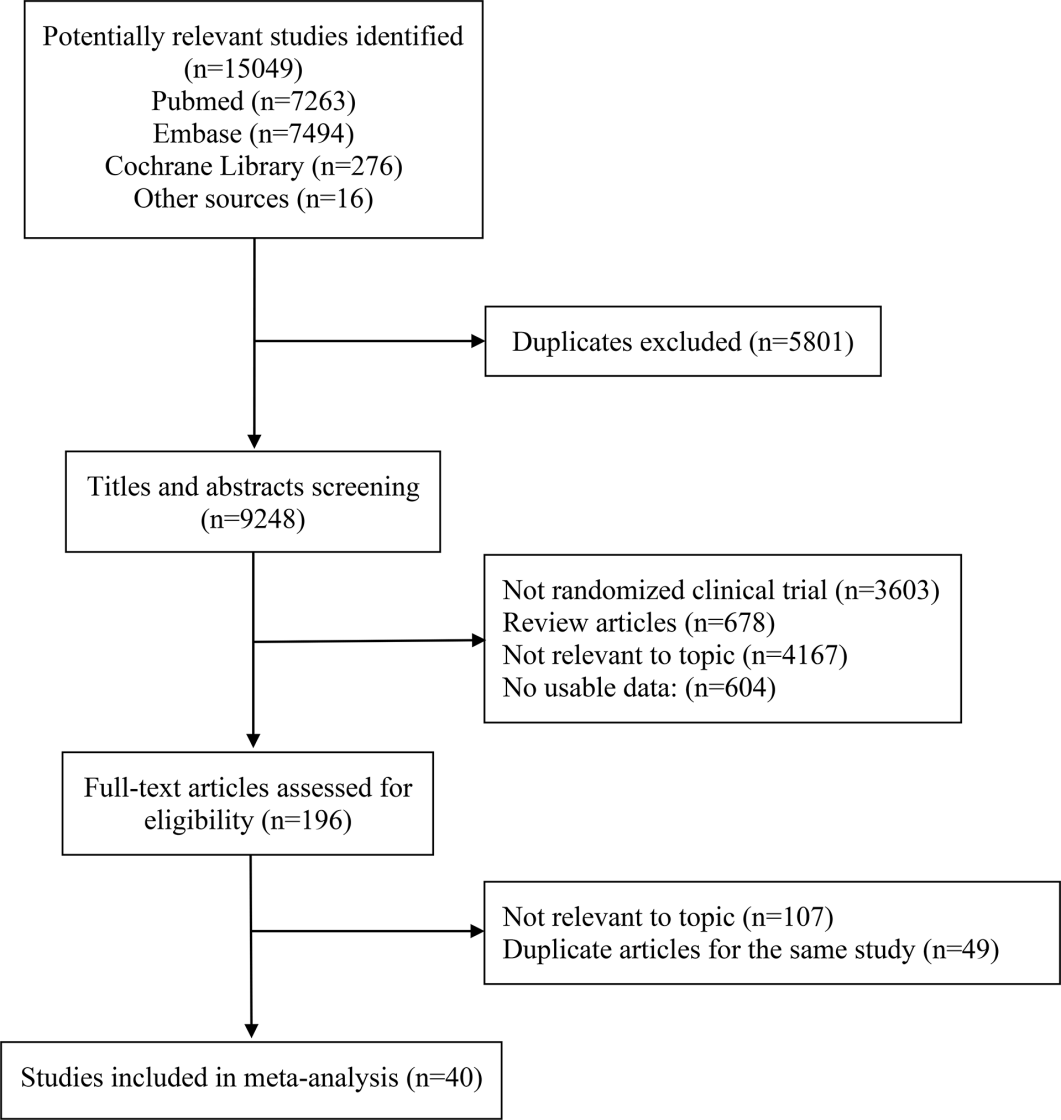
PRISMA flow diagram of randomized controlled trials comparing immune checkpoint inhibitors with chemotherapy.

The literature search retrieved 15049 publications. After detailed evaluation of the abstracts and full texts, 40 eligible RCTs involving 24479 patients were finally included (**Figure 1**). The main characteristics of the included studies are presented in **Table 1**. There were 3 phase II trials, 36 phase III trials, and 1 phase II–III trial. The included studies covered 15 trials comparing ICI monotherapy with CT, 22 trials comparing ICI + CT with CT, which included 2 trials combining ICI with antiangiogenic agents (IMpower150 (15) and ORIENT-31 (57)), 1 trial comparing dual ICIs + CT with CT and the remaining 2 trials had two or three intervention arms (CheckMate 227 (16) and MYSTIC (58)). IMpower150 compared atezolizumab plus chemotherapy, or atezolizumab plus bevacizumab and chemotherapy versus bevacizumab plus chemotherapy, while ORIENT-31 was a study of sintilimab with or without IBI305 (bevacizumab biosimilar) plus chemotherapy versus chemotherapy alone. CheckMate 227 had three parts: in part 1a, patients were randomized to receive nivolumab plus ipilimumab, nivolumab monotherapy, or chemotherapy; in part 1b, patients were randomized to receive nivolumab plus ipilimumab, nivolumab plus chemotherapy, or chemotherapy alone; in part 2, patients were randomized to receive nivolumab plus chemotherapy or chemotherapy alone. MYSTIC randomized patients to receive durvalumab, durvalumab plus tremelimumab, or chemotherapy alone. The dual-ICI arm of MYSTIC was excluded because it didn’t report HRs according to histological type. Ten trials focused on PD-L1 inhibitors, 28 trials on PD-1 inhibitors and 2 trials on a CTLA-4 inhibitor. Nine trials were performed in patients with SQ-NSCLC, 13 trials in those with non-SQ-NSCLC and 18 trials in patients with mixed histological subtypes. All trials were done in advanced or metastatic settings.

**Table 1:** Main characteristics of the included trials.

Study	Year	Phase	Regimen	Line of therapy	Intervention (No.)	Control (No.)	Stage	Histology	PD-L1 IHC Assay	Jadad score	Class of ICI
KEYNOTE-042 (30)	2019	III	ICI	First	Pembrolizumab (637)	Carboplatin + pemetrexed/paclitaxel(637)	III-IV	Mixed	Dako 22C3 pharmDx assay	3	PD-1
KEYNOTE-042 China extension (31)	2020	III	ICI	First	Pembrolizumab (128)	Carboplatin + pemetrexed/paclitaxel (134)	III-IV	Mixed	Dako 22C3 pharmDx assay	3	PD-1
KEYNOTE-024 (8)	2016	III	ICI	First	Pembrolizumab (154)	Carboplatin/cisplatin + pemetrexed/gemcitabine/paclitaxel (151)	IV	Mixed	Dako 22C3 pharmDx assay	3	PD-1
IMpower110 (9)	2020	III	ICI	First	Atezolizumab (277)	Carboplatin/cisplatin + pemetrexed/gemcitabine (277)	IV	Mixed	Ventana SP142 assay	3	PD-L1
CheckMate 026 (32)	2017	III	ICI	First	Nivolumab (271)	Carboplatin/cisplatin + pemetrexed/gemcitabine/paclitaxel (270)	IV	Mixed	Dako 28-8 pharmDx assay	3	PD-1
EMPOWER-Lung 1 (33)	2021	III	ICI	First	Cemiplimab (356)	Carboplatin/cisplatin + pemetrexed/gemcitabine/paclitaxel (354)	IIIB–IV	Mixed	Dako 22C3 pharmDx assay	3	PD-1
POPLAR (7)	2016	II	ICI	Second or later	Atezolizumab (144)	Docetaxel (143)	III-IV	Mixed	Ventana SP142 assay	3	PD-L1
OAK (10)	2017	III	ICI	Second or later	Atezolizumab (613)	Docetaxel (612)	IIIB-IV	Mixed	Ventana SP142 assay	3	PD-L1
KEYNOTE-010 (34)	2016	II/III	ICI	Second or later	Pembrolizumab (690)	Docetaxel (343)	IIIB-IV	Mixed	Dako 22C3 pharmDx assay	3	PD-1
CheckMate 078 (35)	2019	III	ICI	Second or later	Nivolumab (338)	Docetaxel (166)	IIIB-IV	Mixed	Dako 28-8 pharmDx assay	3	PD-1
JAVELIN Lung 200 (36)	2018	III	ICI	Second or later	Avelumab (396)	Docetaxel (396)	IIIB–IV	Mixed	Dako 73-10 pharmDx assay	3	PD-L1
ORIENT-3 (37)	2021	III	ICI	Second or later	Sintilimab (145)	Docetaxel (145)	IIIB–IV	Squamous	Dako 22C3 pharmDx assay	3	PD-1
RATIONALE-303 (38)	2021	III	ICI	Second or later	Tislelizumab (535)	Docetaxel (270)	IIIB–IV	Mixed	Ventana SP263 assay	3	PD-1
CheckMate 057 (5)	2015	III	ICI	Second or later	Nivolumab (292)	Docetaxel (290)	IIIB-IV	Non-squamous	Dako 28-8 pharmDx assay	3	PD-1
CheckMate 017 (6)	2015	III	ICI	Second or later	Nivolumab (135)	Docetaxel (137)	IIIB–IV	Squamous	Dako 28-8 pharmDx assay	3	PD-1
IMpower130 (39)	2019	III	ICI + CT	First	Atezolizumab +carboplatin + nab-paclitaxel (483)	Carboplatin + nab-paclitaxel (240)	IV	Non-squamous	Ventana SP142 assay	3	PD-L1
IMpower150 (ABCP) (15)	2018	III	ICI + CT	First	Atezolizumab + bevacizumab + carboplatin + paclitaxel (400)	Bevacizumab + carboplatin + paclitaxel (400)	IV	Non-squamous	Ventana SP142 assay	3	PD-L1
IMpower150 (ACP) (15)	2018	III	ICI + CT	First	Atezolizumab + carboplatin + paclitaxel (402)	Bevacizumab + carboplatin + paclitaxel (400)	IV	Non-squamous	Ventana SP142 assay	3	PD-L1
KEYNOTE-189 (12)	2018	III	ICI + CT	First	Pembrolizumab + carboplatin/cisplatin + pemetrexed (410)	Carboplatin/cisplatin + pemetrexed (206)	IV	Non-squamous	Agilent 22C3 pharmDx assay	5	PD-1
KEYNOTE-189 Japan extension (40)	2021	III	ICI + CT	First	Pembrolizumab + carboplatin/cisplatin + pemetrexed (25)	Carboplatin/cisplatin + pemetrexed (15)	IV	Non-squamous	Agilent 22C3 pharmDx assay	5	PD-1
KEYNOTE-021 (13)	2016	II	ICI + CT	First	Pembrolizumab + carboplatin + pemetrexed (60)	Carboplatin + pemetrexed (63)	IIIB-IV	Non-squamous	Dako 22C3 pharmDx assay	3	PD-1
IMpower132 (41)	2020	III	ICI + CT	First	Atezolizumab + carboplatin/cisplatin + pemetrexed (292)	Carboplatin/cisplatin + pemetrexed (286)	IV	Non-squamous	Ventana SP142 assay	3	PD-L1
CameL (42)	2020	III	ICI + CT	First	Camrelizumab + carboplatin + pemetrexed (205)	Carboplatin + pemetrexed (207)	IIIB–IV	Non-squamous	Dako 22C3 pharmDx assay	5	PD-1
TASUKI-52 (43)	2021	III	ICI + CT	First	Nivolumab + carboplatin + paclitaxel + bevacizumab (275)	Carboplatin + paclitaxel + bevacizumab (275)	IIIB–IV	Non-squamous	Dako 28-8 pharmDx assay	5	PD-1
RATIONALE 304 (44)	2021	III	ICI + CT	First	Tislelizumab + carboplatin/cisplatin + pemetrexed (223)	Carboplatin/cisplatin + pemetrexed (111)	IIIB–IV	Non-squamous	Ventana SP263 assay	3	PD-1
ORIENT-11 (45)	2020	III	ICI + CT	First	Sintilimab + carboplatin/cisplatin + pemetrexed (266)	Carboplatin/cisplatin + pemetrexed (131)	IIIB–IV	Non-squamous	Dako 22C3 pharmDx assay	5	PD-1
KEYNOTE-407 (14)	2020	III	ICI + CT	First	Pembrolizumab + carboplatin + paclitaxel/nab-paclitaxel (278)	Carboplatin + paclitaxel/nab-paclitaxel (281)	IV	Squamous	Agilent 22C3 pharmDx assay	5	PD-1
KEYNOTE-407 China extension (46)	2021	III	ICI + CT	First	Pembrolizumab + carboplatin + paclitaxel/nab-paclitaxel (63)	Carboplatin + paclitaxel/nab-paclitaxel (60)	IV	Squamous	Agilent 22C3 pharmDx assay	5	PD-1
IMpower131 (47)	2020	III	ICI + CT	First	Atezolizumab + carboplatin + nab-paclitaxel (343)	Carboplatin + nab-paclitaxel (340)	IV	Squamous	Ventana SP142 assay	3	PD-L1
RATIONALE 307 (P) (48)	2021	III	ICI + CT	First	Tislelizumab + carboplatin + paclitaxel (120)	Carboplatin + paclitaxel (121)	IIIB–IV	Squamous	Ventana SP263 assay	3	PD-1
RATIONALE 307 (NP) (48)	2021	III	ICI + CT	First	Tislelizumab + carboplatin + nab-paclitaxel (119)	Carboplatin + paclitaxel (121)	IIIB–IV	Squamous	Ventana SP263 assay	3	PD-1
ORIENT-12 (49)	2021	III	ICI + CT	First	Sintilimab + carboplatin/cisplatin +gemcitabine (179)	Carboplatin/cisplatin + gemcitabine (178)	IIIB–IV	Squamous	Dako 22C3 pharmDx assay	5	PD-1
CameL-sq (50)	2021	III	ICI + CT	First	Camrelizumab + carboplatin + paclitaxel (193)	Carboplatin + paclitaxel (196)	IIIB–IV	Squamous	Dako 22C3 pharmDx assay	5	PD-1
CheckMate 227 Part-2 (51)	2019	III	ICI + CT	First	Nivolumab + carboplatin/cisplatin + gemcitabine/pemetrexed (377)	Carboplatin/cisplatin + gemcitabine/pemetrexed (378)	IV	Mixed	Dako 28-8 pharmDx assay	3	PD-1
CHOICE-01 (52)	2021	III	ICI + CT	First	Toripalimab + carboplatin/cisplatin + paclitaxel/pemetrexed (309)	Carboplatin/cisplatin + paclitaxel/pemetrexed (156)	III	Mixed	–	5	PD-1
GEMSTONE-302 (53)	2021	III	ICI + CT	First	Sugemalimab + carboplatin + paclitaxel/pemetrexed (320)	Carboplatin + paclitaxel/pemetrexed (159)	IV	Mixed	Ventana SP263 assay	5	PD-L1
Govindan (54)	2017	III	ICI + CT	First	Ipilimumab + carboplatin + paclitaxel (388)	Carboplatin + paclitaxel (361)	IV	Squamous	NA	5	CTLA-4
Lynch (C) (55)	2012	III	ICI + CT	First	Ipilimumab + paclitaxel + carboplatin (concurrent) (70)	Carboplatin + paclitaxel (66)	IIIB–IV	Mixed	NA	5	CTLA-4
Lynch (P) (55)	2012	III	ICI + CT	First	Ipilimumab + carboplatin + paclitaxel (phased) (68)	Carboplatin + paclitaxel (66)	IIIB–IV	Mixed	NA	5	CTLA-4
PROLUNG (56)	2020	II	ICI + CT	Second or later	Pembrolizumab + docetaxel (40)	Docetaxel (38)	IV	Non-squamous	Dako 22C3 pharmDx assay	3	PD-1
ORIENT-31 (S + IBI305) (57)	2021	III	ICI + CT	Second or later	Sintilimab + IBI305 + cisplatin + pemetrexed (148)	Cisplatin + pemetrexed (151)	III-IV	Non-squamous	Dako 22C3 pharmDx assay	5	PD-1
ORIENT-31 (S) (57)	2021	III	ICI + CT	Second or later	Sintilimab + cisplatin + pemetrexed (145)	Cisplatin + pemetrexed (151)	III-IV	Non-squamous	Dako 22C3 pharmDx assay	5	PD-1
MYSTIC (D) (58)	2020	III	ICI	First	Durvalumab (374)	Carboplatin/cisplatin + pemetrexed/gemcitabine/paclitaxel (372)	IV	Mixed	Ventana SP263 assay	3	PD-L1
CheckMate 227 Part-1a (N) (16)	2019	III	ICI	First	Nivolumab (396)	Carboplatin/cisplatin + pemetrexed/gemcitabine (397)	IV	Mixed	Dako 28-8 pharmDx assay	3	PD-1
CheckMate 227 Part-1a (N + I) (16)	2019	III	ICI + ICI	First	Nivolumab + ipilimumab (583)	Carboplatin/cisplatin + gemcitabine/pemetrexed (583)	IV	Mixed	Dako 28-8 pharmDx assay	3	PD-1 + CTLA-4
CheckMate 227 Part-1b (16)	2019	III	ICI + CT	First	Nivolumab + carboplatin/cisplatin + gemcitabine/pemetrexed (177)	Carboplatin/cisplatin + gemcitabine/pemetrexed (186)	IV	Mixed	Dako 28-8 pharmDx assay	3	PD-1
CheckMate 9LA (17)	2021	III	ICI + ICI +CT	First	Nivolumab + ipilimumab + carboplatin/cisplatin + paclitaxel/pemetrexed (361)	Carboplatin/cisplatin + paclitaxel/pemetrexed (358)	IV	Mixed	Dako 28-8 pharmDx assay	3	PD-1 + CTLA-4

ABCP, atezolizumab in combination with bevacizumab plus carboplatin plus paclitaxel; ACP, atezolizumab plus carboplatin plus paclitaxel; P, paclitaxel; NP, nab-paclitaxel; Lynch (C), a concurrent ipilimumab regimen; Lynch (P), a phased ipilimumab regimen; S + IBI305, sintilimab + IBI305; S, sintilimab; D, durvalumab; N, nivolumab; N + I, nivolumab + ipilimumab; ICI, immune checkpoint inhibitor; CT, chemotherapy; PD-1, programmed cell death 1; PD-L1, programmed death-ligand 1; CTLA-4, cytotoxic T lymphocyte-associated protein-4.

### Survival benefits of ICIs compared with chemotherapy in SQ-NSCLC and non-SQ-NSCLC

Meta-analyses of all included studies demonstrated that ICI monotherapy could improve OS regardless of line of treatment in both SQ-NSCLC and non-SQ-NSCLC (OS-HR 0.71, 95% CI 0.65-0.77 and OS-HR 0.80, 95% CI 0.73-0.87, respectively) while the OS benefit was smaller in non-SQ-NSCLC (**Supplementary Figure 1** and **Table 2**). In terms of PFS, ICI monotherapy could reduce the risk of progression by 35% (PFS-HR 0.65, 95% CI 0.56-0.77) in SQ-NSCLC while the PFS benefit was smaller (10%) and not statistically significant in non-SQ-NSCLC (PFS-HR 0.90, 95% CI 0.76-1.07; **Supplementary Figure 2**, **Table 2**).

ICI-based combination treatments could reduce the risk of both progression and death in SQ-NSCLC (OS-HR 0.70, 95% CI 0.61-0.80; PFS-HR 0.56, 95% CI 0.48-0.65) and non-SQ-NSCLC (OS-HR 0.78, 95% CI 0.74-0.83; PFS-HR 0.63, 95% CI 0.57-0.69) regardless of combination modalities (ICI + CT, dual ICIs, or dual ICIs + CT) while the survival benefits were more pronounced in SQ-NSCLC (**Supplementary Figures 3**-**4**, **Table 2**).

Substantial inter-study heterogeneity was observed in both SQ-NSCLC (OS: I (2) = 29.1%, P = 0.077; PFS: I (2) = 73.1%, P <0.001) and non-SQ-NSCLC (OS: I (2) = 39.3%, P = 0.013; PFS: I (2) = 80.8%, P <0.001). Sensitivity analysis by removing studies that enrolled patients with *EGFR* or *ALK* mutations (CheckMate 057 (5), OAK (10), POPLAR (7), KEYNOTE-010 (34), IMpower150 (15), IMpower130 (39), PROLUNG (56), and ORIENT-31 (57)) did not alter the results (**Supplementary Figures 5**-**8**).

The results of subgroup analyses stratified by the class of ICI are shown in **Table 2**. PD-1 inhibitors could improve OS and PFS in both SQ-NSCLC (OS-HR 0.67, 95% CI 0.62-0.72; PFS-HR 0.58, 95% CI 0.52-0.65) and non-SQ-NSCLC (OS-HR 0.77, 95% CI 0.71-0.84; PFS-HR 0.67, 95% CI 0.59-0.76). Similarly, PD-L1 inhibitors could improve OS and PFS in both SQ-NSCLC (OS-HR 0.82, 95% CI 0.73-0.93; PFS-HR 0.55, 95% CI 0.35-0.86) and non-SQ-NSCLC (OS-HR 0.81, 95% CI 0.76-0.87; PFS-HR 0.72, 95% CI 0.57-0.90). However, CTLA-4 inhibitor (ipilimumab) could only reduce the risk of progression in SQ-NSCLC and it offered no survival benefit in non-SQ-NSCLC.

**Table 2:** Pooled HRs and pooled ratios of HRs for OS and PFS by subgroups.

	SQ-NSCLC			non-SQ-NSCLC							RHR (95% CI)							P_interaction_
	Studies, n	Patients, n	HR (95% CI)	Studies, n		Patients, n				HR (95% CI)								
***Overall survival***																		
**All included studies**																		
Overall	28	7547	0.73 (0.69-0.77)	32		14578	0.79 (0.75-0.84)							–				–
ICI monotherapy	16	3763	0.71 (0.65-0.77)	15		7109	0.80 (0.73-0.87)							–				–
ICI-based combination treatments	12	3784	0.70 (0.61-0.80)	17		7469	0.78 (0.74-0.83)							–				–
Class of ICI																		
anti-PD-1	19	5000	0.67 (0.62-0.72)	21		8916	0.77 (0.71-0.84)							–				–
anti-PD-L1	6	1726	0.82 (0.73-0.93)	9		5464	0.81 (0.76-0.87)							–				–
anti-CTLA-4	3	821	0.89 (0.76-1.04)	2		198	1.06 (0.76-1.47)							–				–
**Studies with paired subgroups**																		
Overall	21	4395	0.70 (0.65-0.75)	21		9459		0.80 (0.76-0.84)				0.88 (0.80-0.96)					0.004	
ICI monotherapy	15	3473	0.71 (0.65-0.77)	15		7109		0.80 (0.73-0.87)				0.89 (0.80-0.99)					0.031	
ICI-based combination treatments	6	922	0.67 (0.58-0.78)	6		2350		0.81 (0.74-0.89)				0.83 (0.70-0.99)					0.040	
Class of ICI																		
anti-PD-1	14	3280	0.68 (0.62-0.73)	14		6700		0.80 (0.75-0.85)				0.85 (0.77-0.94)					0.002	
anti-PD-L1	5	1043	0.78 (0.67-0.92)	5		2361		0.79 (0.71-0.88)				1.00 (0.82-1.21)					0.976	
anti-CTLA-4	2	72	0.72 (0.43-1.22)	2		198		1.06 (0.76-1.47)				0.67 (0.36-1.25)					0.132	
***Progression-free survival***																		
**All included studies**																		
Overall	25	6630	0.59 (0.53-0.66)	30		12696		0.69 (0.62-0.77)				–			–			
ICI monotherapy	9	1953	0.65 (0.56-0.77)	8		3766		0.90 (0.76-1.07)				–			–			
ICI-based combination treatments	16	4677	0.56 (0.48-0.65)	22		8930		0.63 (0.57-0.69)				–			–			
Class of ICI																		
anti-PD-1	19	4692	0.58 (0.52-0.65)	22		8758		0.67 (0.59-0.76)				–			–			
anti-PD-L1	3	1117	0.55 (0.35-0.86)	6		3740		0.72 (0.57-0.90)				–			–			
anti-CTLA-4	3	821	0.85 (0.73-0.98)	2		198		0.84 (0.62-1.15)				–			–			
**Studies with paired subgroups**																		
Overall	16	2997	0.63 (0.55-0.72)	16	6648				0.81 (0.72-0.91)				0.79 (0.71-0.87)			<0.001		
ICI monotherapy	8	1663	0.69 (0.62-0.77)	8	3766				0.90 (0.76-1.07)				0.76 (0.65-0.88)			<0.001		
ICI-based combination treatments	8	1334	0.58 (0.46-0.72)	8	2882				0.72 (0.65-0.79)				0.82 (0.70-0.96)			0.016		
Class of ICI																		
anti-PD-1	12	2491	0.66 (0.58-0.75)	12	5613				0.79 (0.70-0.90)				0.82 (0.73-0.93)			0.001		
anti-PD-L1	2	434	0.47 (0.25-0.88)	2	837				0.87 (0.41-1.86)				0.55 (0.39-0.76)			<0.001		
anti-CTLA-4	2	72	0.60 (0.28-1.28)	2	198				0.84 (0.62-1.15)				0.71 (0.38-1.32)			0.278		

OS, overall survival; PFS, progression-free survival; HR, hazard ratio; NSCLC, non-small-cell lung cancer; SQ-NSCLC, squamous NSCLC; non-SQ-NSCLC, non-squamous NSCLC; CI, confidence interval; RHR, ratio of the HR in SQ-NSCLC to the HR in non-SQ-NSCLC; ICI, immune checkpoint inhibitor; PD-1, programmed cell death 1; PD-L1, programmed death-ligand 1; CTLA-4, cytotoxic T lymphocyte-associated protein-4.

**Figure 2:**
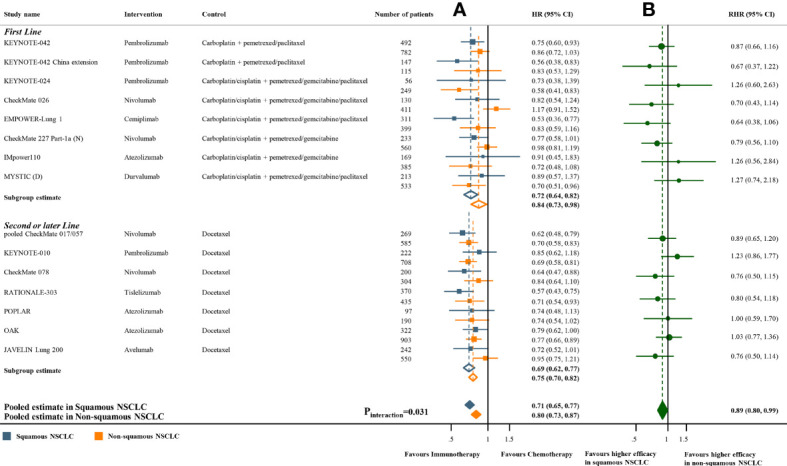
Overall survival benefits of ICI monotherapy compared with chemotherapy in studies with paired subgroups. **(A)** HRs according to histology. **(B)** Trial-specific ratios of the HR in SQ-NSCLC to the HR in non-SQ-NSCLC. ICI, immune checkpoint inhibitor; HR, hazard ratio; RHR, ratios of the HRs; CI, confidence interval.

**Figure 3:**
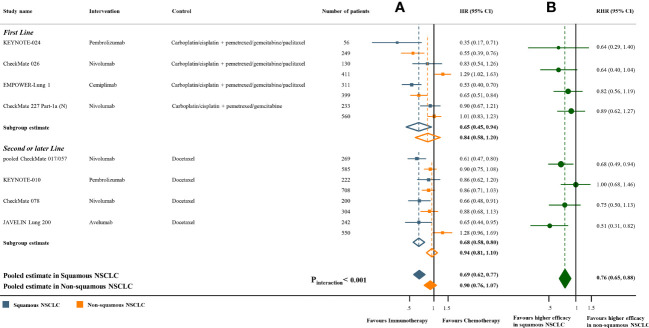
Progression-free survival benefits of ICI monotherapy compared with chemotherapy in studies with paired subgroups. **(A)** HRs according to histology. **(B)** Trial-specific ratios of the HR in SQ-NSCLC to the HR in non-SQ-NSCLC. ICI, immune checkpoint inhibitor; HR, hazard ratio; RHR, ratios of the HRs; CI, confidence interval.

**Figure 4:**
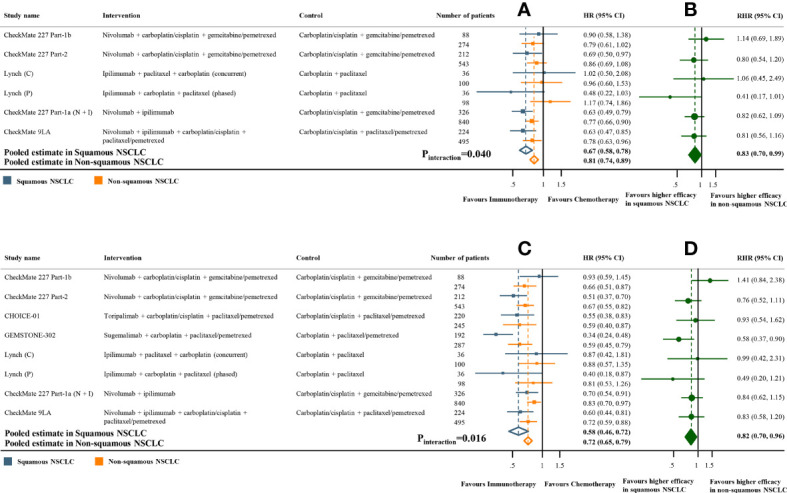
Overall and progression-free survival benefits of ICI-based combination treatments compared with chemotherapy in studies with paired subgroups. **(A)** HRs of overall survival according to histology. **(B)** Trial-specific ratios of the HR in SQ-NSCLC to the HR in non-SQ-NSCLC (RHR). **(C)** HRs of progression-free survival according to histology. **(D)** Trial-specific ratios of the HR in SQ-NSCLC to the HR in non-SQ-NSCLC. ICI, immune checkpoint inhibitor; HR, hazard ratio; RHR, ratios of the HRs; CI, confidence interval.

Meta-analyses of the studies with paired subgroups showed consistent results. ICI monotherapy could improve OS in both SQ-NSCLC (OS-HR 0.71, 95% CI 0.65-0.77) and non-SQ-NSCLC (OS-HR 0.80, 95% CI 0.73-0.87) while the OS benefit was smaller in non-SQ-NSCLC (**Figure 2A**). PFS benefit was observed in SQ-NSCLC (PFS-HR 0.69, 95% CI 0.62-0.77), but it was smaller and not statistically significant in non-SQ-NSCLC (PFS-HR 0.90, 95% CI 0.76-1.07) when ICIs were given alone (**Figure 3A**). ICI-based combination treatments could reduce the risk of both progression and death in SQ-NSCLC (OS-HR 0.67, 95% CI 0.58-0.78; PFS-HR 0.58, 95% CI 0.46-0.72) and non-SQ-NSCLC (OS-HR 0.81, 95% CI 0.74-0.89; PFS-HR 0.72, 95% CI 0.65-0.79) while the survival benefits were more pronounced in SQ-NSCLC (**Figures 4A**, **4C**). Subgroup analysis stratified by the class of ICI demonstrated that PD-1 inhibitors could improve OS and PFS in both histological subtypes, with larger benefits in SQ-NSCLC (**Supplementary Figures 9A**, **10A**), while PD-L1 inhibitors could not improve PFS in non-SQ-NSCLC (PFS-HR 0.87, 95% CI 0.41-1.86).

### Effect of histology on the efficacy of ICIs

The above analyses demonstrated that SQ-NSCLC could obtain more survival benefits from ICIs, although the risk of progression and death decreased in both SQ-NSCLC and non-SQ-NSCLC due to the addition of ICIs. Next, we further evaluated the effect of histology on the efficacy of ICIs.

The pooled ratio of OS-HRs reported in SQ-NSCLC versus OS-HRs reported in non-SQ-NSCLC (OS-RHR) in each trial evaluating the survival benefit of ICI monotherapy was 0.89 (95% CI 0.80-0.99, P_interaction_ =0.031; **Figure 2B**). Similarly, the pooled PFS-RHR was 0.76 (95% CI 0.65-0.88, P_interaction_ <0.001; **Figure 3B**). These results indicated a significantly larger survival benefit of ICI monotherapy in SQ-NSCLC compared with non-SQ-NSCLC. As regards ICI-based combination treatments, the pooled OS-RHR was 0.83 (95% CI 0.70-0.99, P_interaction_ =0.040; **Figure 4B**) and the pooled PFS-RHR was 0.82 (95% CI 0.70-0.96, P_interaction_ =0.016; **Figure 4D**), suggesting that the efficacy of ICI-based combination treatments in SQ-NSCLC was higher than that in non-SQ-NSCLC.

Sensitivity analysis was conducted by removing studies that enrolled patients with *EGFR* or *ALK* mutations. Only 4 studies with paired subgroups enrolled patients with *EGFR* or *ALK* mutations (CheckMate 057 (5), OAK (10), POPLAR (7) and KEYNOTE-010 (34)) and all these studies compared ICI monotherapy with chemotherapy. After removing these studies, the results remained unchanged (**Supplementary Figures 11**-**12**). Subgroup analysis stratified by the class of ICI showed that SQ-NSCLC could derive significantly more survival benefits from PD-1 inhibitors compared to non-SQ-NSCLC (OS-RHR 0.85, 95% CI 0.77-0.94; PFS-RHR 0.82, 95% CI 0.73-0.93; **Supplementary Figures 9B** and **10B**). However, no significant difference in the efficacy of PD-L1 inhibitors regarding OS was noted between SQ-NSCLC and non-SQ-NSCLC (OS-RHR 1.00, 95% CI 0.82-1.21; **Supplementary Figures 9B**). Regarding the CTLA-4 inhibitor (ipilimumab), no significant difference in the efficacy of ipilimumab between SQ-NSCLC and non-SQ-NSCLC was observed.

### Quality assessment of the studies and publication bias

Jadad scores for each trial are summarized in **Table 1**. All trials had moderate-to-high quality (Jadad scores of 3–5). The Egger’s test yielded no publication bias in OS (SQ-NSCLC: P= 0.090; non-SQ-NSCLC: P= 0.951) and PFS (SQ-NSCLC: P= 0.115; non-SQ-NSCLC: P= 0.250).

## Discussion

Although ICIs have been approved for the treatment of patients with advanced NSCLC, regardless of histology, whether they deliver different clinical efficacy in SQ-NSCLC and non-SQ-NSCLC remains controversial (22). The survival benefits were larger in SQ-NSCLC compared with non-SQ-NSCLC in some trials (KEYNOTE-042 (30), CheckMate 026 (32), CheckMate 227 (16), CheckMate 078 (35)), and were similar in OAK (10), POPLAR (7) and CHOICE-01 (52), but were smaller in KEYNOTE-010 (34) and KEYNOTE-024 (8). To investigate if there is a difference in the efficacy of ICIs between SQ-NSCLC and non-SQ-NSCLC, we conducted this systematic review and meta-analysis.

The results demonstrated that ICIs could improve OS and PFS in both SQ-NSCLC and non-SQ-NSCLC while SQ-NSCLC derived significantly larger survival benefits from ICIs (vs CT) as compared with non-SQ-NSCLC. As a strength of this work, the effect of histology on the efficacy of ICIs was assessed separately for each type of immunotherapeutic strategy (ICI monotherapy and ICI-based combination treatments), considering different underlying mechanisms. ICI monotherapy could reduce the risk of death by 29% in SQ-NSCLC and 20% in non-SQ-NSCLC and the OS-RHR was 0.89 (P_interaction_ =0.031), indicating higher efficacy in SQ-NSCLC. In terms of PFS, ICI monotherapy could reduce the risk of progression by 35% in SQ-NSCLC while the PFS benefit was smaller (10%) and not statistically significant in non-SQ-NSCLC. Similarly, ICI-based combination treatments could reduce the risk of both progression and death in SQ-NSCLC and non-SQ-NSCLC while the survival benefits were more pronounced in SQ-NSCLC.

SQ-NSCLC and non-SQ-NSCLC are two different biologic subtypes with distinct genomic alterations and immune microenvironment (22–24). More extensive infiltration of CD8^+^ effector T cells and less profound accumulation of Foxp3^+^ regulatory T cells were observed in SQ-NSCLC as compared to non-SQ-NSCLC (23, 59), which partly explained the higher efficacy of ICIs in SQ-NSCLC. Besides, SQ-NSCLC is associated with a higher likelihood of smoking. Studies have shown that SQ-NSCLC was more frequently observed in smokers while adenocarcinoma was more prevalent among never smokers (60, 61). Smokers are generally correlated with higher levels of mutational burden, neoantigens, and PD-L1 expression and thereby may obtain greater benefit from ICIs (62, 63). A recently published study confirmed that ICIs were associated with significant longer OS and PFS than chemotherapy in smokers but not in never smokers (64). Consistently, studies have demonstrated that TMB and neoantigens are significantly higher in SQ-NSCLC compared with adenocarcinoma (65), with this being another potential biological rationale to explain the higher efficacy in SQ-NSCLC. Furthermore, oncogenic driver mutations, such as *EGFR* mutation and *ALK* translocation, are more common in adenocarcinoma than in SQ-NSCLC (2, 22). *EGFR* mutation is associated with an uninflamed tumour microenvironment and low TMB, leading to less sensitivity to ICI (66, 67). *ALK* translocation is also considered a negative predictive factor of response to ICI (67). To assess the stability of our results, sensitivity analyses were conducted by excluding studies that enrolled patients with *EGFR* or *ALK* mutations. The results remained unchanged after sensitivity analyses, confirming the higher efficacy of ICIs in SQ-NSCLC.

PD-1 inhibitors were reported to exhibit superior efficacy compared with PD-L1 inhibitors either as monotherapy or in combination with chemotherapy in NSCLC (68). In the present study, the pooled HRs in patients receiving PD-1 inhibitors were smaller than those in patients receiving PD-L1 inhibitors (**Table 2**), indirectly indicating that PD-1 inhibitors could deliver more pronounced survival benefits. Besides, in studies with paired subgroups, we found that PD-1 inhibitors could improve PFS while the PFS benefit of PD-L1 inhibitors was not statistically significant in non-SQ-NSCLC, also indicating better efficacy of PD-1 inhibitors. This study also suggested that PD-1 inhibitors delivered more pronounced OS benefits in SQ-NSCLC compared with non-SQ-NSCLC while no significant difference in the efficacy of PD-L1 inhibitors regarding OS was noted between SQ-NSCLC and non-SQ-NSCLC. One main reason for the discrepancy in the effect of histology on the efficacy of PD-1 inhibitors and PD-L1 inhibitors is the inherent differences between these two inhibitors. Theoretically, PD-L1 inhibitors only inhibit the binding of PD-1 to PD-L1. No difference in PD-L1 expression was observed between SQ-NSCLC and non-SQ-NSCLC (12, 14, 23). Thus, SQ-NSCLC and non-SQ-NSCLC may obtain comparable survival benefits from PD-L1 inhibitors. In contrast, PD-1 inhibitors can bind to PD-1 on T cells, so it will block the binding of PD-1 to PD-L1 and programmed death ligand 2 (PD-L2) at the same time. PD-1 and PD-L2 expression levels were reported to be significantly higher in SQ-NSCLC (69). Besides, PD-L2 expression level was also identified as a predictive biomarker of survival benefit from ICI (70). Therefore, SQ-NSCLC could derive larger survival benefits from PD-1 inhibitors compared to non-SQ-NSCLC.

To our knowledge, this is the first meta-analysis to clearly show the effect of histology on the efficacy of ICIs in NSCLC. One previous meta-analysis by Lee et al (71) showed that there was no difference in survival benefits from ICI between SQ-NSCLC and non-SQ-NSCLC. However, the reliability of the study may be inconclusive in several ways. First, the result was based on only two studies, which may lead to a high false discovery rate. Second, the comparison of survival benefits from ICIs in SQ-NSCLC vs survival benefits in non-SQ-NSCLC was conducted only between the pooled HRs on each side, leaving the important issue of ecological bias or confounding (72). In our study, to avoid the risk of ecological bias, we first calculated an interaction trial-specific RHR (ratio of the HR in SQ-NSCLC to the HR in non-SQ-NSCLC) for each trial with paired subgroups, which ensured the comparability of the HRs in SQ-NSCLC and non-SQ-NSCLC. Then we combined these trial-specific RHRs across trials to obtain a pooled RHR. Another meta-analysis investigating the survival benefits of ICIs according to the histology also suggested that ICI monotherapy significantly prolonged OS in both SQ-NSCLC and non-SQ-NSCLC (73). However, the effect of histology on the efficacy of ICIs was not assessed and it didn’t evaluate the survival benefits of ICI-based combination treatments. Other strengths of this meta-analysis include the broad inclusion of all recently published RCTs, the strict method-logic inclusion criteria and rigorous statistical analyses. As a result, this study provides a comprehensive assessment of the difference in the efficacy of ICIs between SQ-NSCLC and non-SQ-NSCLC.

This study has several limitations. First, this meta-analysis relied on trial-level results and not on individual patient-level data. Second, not all included trials provided paired subgroups according to histology. Third, there was substantial inter-study heterogeneity although the random-effects model was used to minimize its influence and results were consistent across sensitivity analyses and subgroup analyses. Further studies are warranted to confirm our findings.

In conclusion, ICIs could deliver survival benefits in both SQ-NSCLC and non-SQ-NSCLC while the magnitude of survival benefits was histology-dependent. SQ-NSCLC derived larger survival benefits from ICIs, especially PD-1 inhibitors, as compared with non-SQ-NSCLC. The effect of histology on the efficacy of ICIs should be taken into account in future researches and clinical practice.

## Data availability statement

The raw data supporting the conclusions of this article will be made available by the authors, without undue reservation.

## Author contributions

FL: Conceptualization, Data curation, Methodology, Formal analysis, Writing—original draft; SZ: Conceptualization, Data curation, Writing—review & editing; ZL: Data curation, Formal analysis, Visualisation, Writing—original draft; LY: Methodology, Formal analysis; SW: Methodology, Formal analysis; DJ: Methodology, Formal analysis; HY: Methodology, Formal analysis; LF: Methodology, Formal analysis; YM: Conceptualization, Supervision, Writing—review & editing. All authors contributed to the article and approved the submitted version.

## Conflict of interest

The authors declare that the research was conducted in the absence of any commercial or financial relationships that could be construed as a potential conflict of interest.

## Publisher’s note

All claims expressed in this article are solely those of the authors and do not necessarily represent those of their affiliated organizations, or those of the publisher, the editors and the reviewers. Any product that may be evaluated in this article, or claim that may be made by its manufacturer, is not guaranteed or endorsed by the publisher.

## Abbreviations

ICI, immune checkpoint inhibitor; NSCLC, non-small-cell lung cancer; OS, overall survival; CT, chemotherapy; PD-1, programmed cell death 1; PD-L1, programmed death ligand 1; PD-L2, programmed death ligand 2; TMB, tumour mutational burden; CTLA-4, cytotoxic T-cell lymphocyte antigen-4; RCT, randomized clinical trial; SQ-NSCLC, squamous non-small-cell lung cancer; non-SQ-NSCLC, non-squamous non-small-cell lung cancer; PRISMA, Preferred Reporting Items for Systematic Reviews and Meta-analysis; HR, hazard ratio; PFS, progression-free survival; PFS-HR, hazard ratio for progression-free survival; OS-HR, hazard ratio for overall survival; CI, confidence interval; RHR, ratio of hazard ratios; OS-RHR, ratio of hazard ratios for overall survival; PFS-RHR, ratio of hazard ratios for progression-free survival; P_interaction_, P value for interaction; EGFR, epidermal growth factor receptor; ALK, anaplastic lymphoma kinase.
