# Supplementary material for: Effect of histology on the efficacy of immune checkpoint inhibitors in advanced non-small cell lung cancer: A systematic review and meta-analysis
Source: Front Oncol. 2022 Nov 10;12:968517. doi: 10.3389/fonc.2022.968517 (PMC9685340; doi:10.3389/fonc.2022.968517)
Supplement: Supplementary file 1 [file Image_1.pdf]

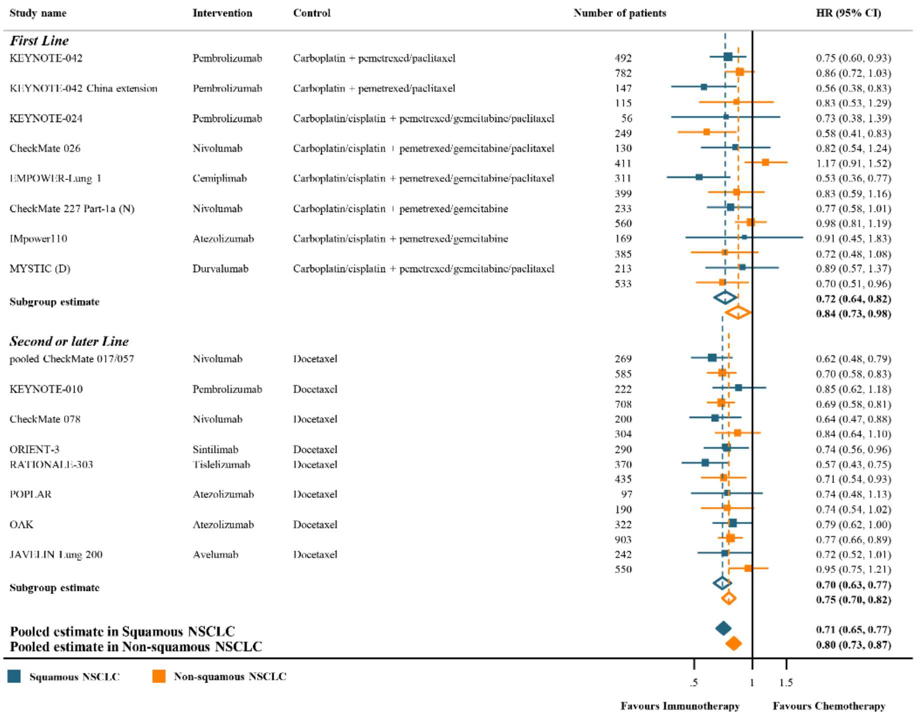

Supplementary Figure 1: Overall survival benefits of ICI monotherapy compared with chemotherapy according to histology in all included studies. ICI, immune checkpoint inhibitor; HR, hazard ratio; RHR, ratios of the HRs; CI, confidence interval.

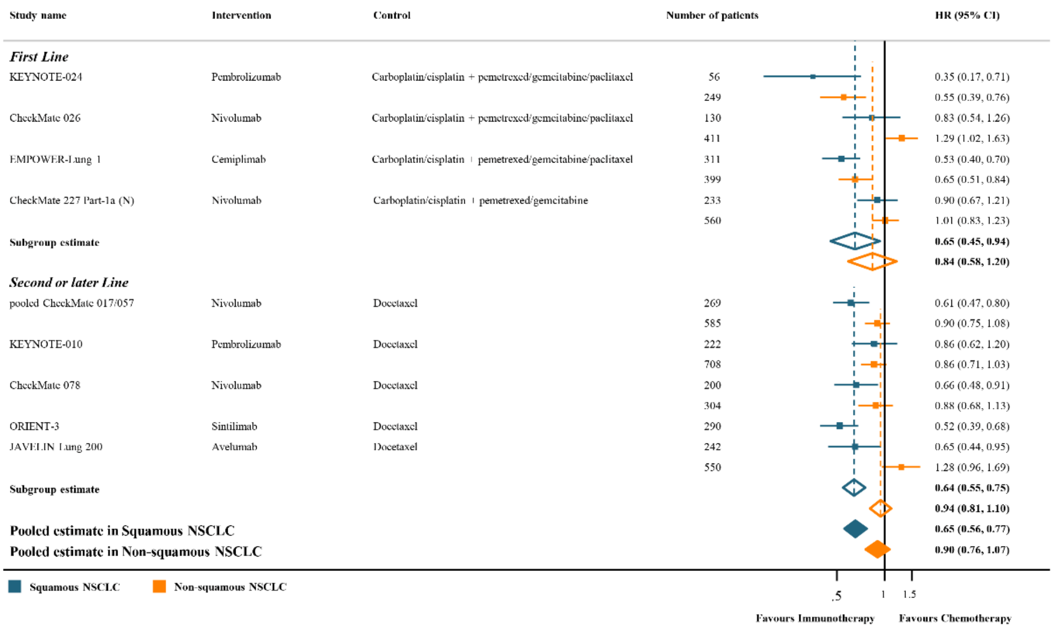

Supplementary Figure 2: Progression-free survival benefits of ICI monotherapy compared with chemotherapy according to histology in all included studies. ICI, immune checkpoint inhibitor; HR, hazard ratio; RHR, ratios of the HRs; CI, confidence interval.

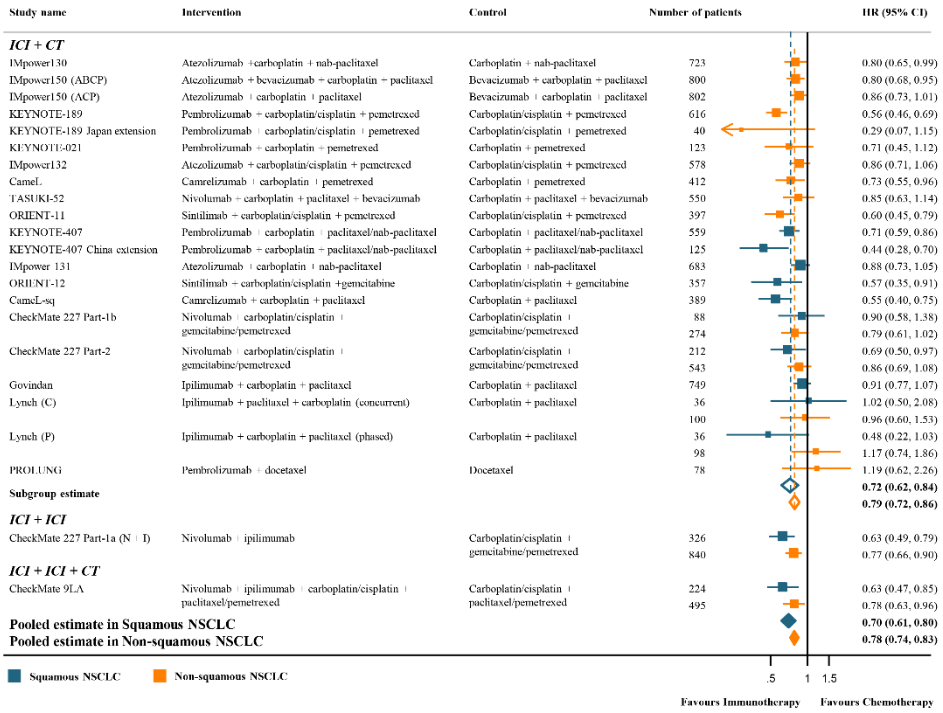

Supplementary Figure 3: Overall survival benefits of ICI-based combination treatments compared with chemotherapy according to histology in all included studies. ICI, immune checkpoint inhibitor; HR, hazard ratio; RHR, ratios of the HRs; CI, confidence interval.

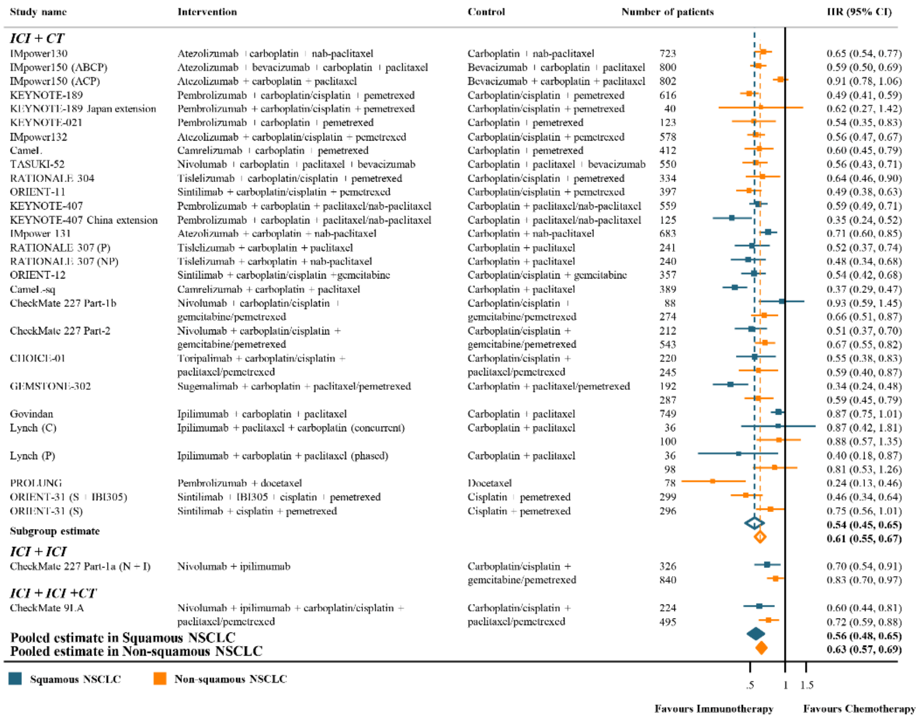

Supplementary Figure 4: Progression-free survival benefits of ICI-based combination treatments compared with chemotherapy according to histology in all included studies. ICI, immune checkpoint inhibitor; HR, hazard ratio; RHR, ratios of the HRs; CI, confidence interval.

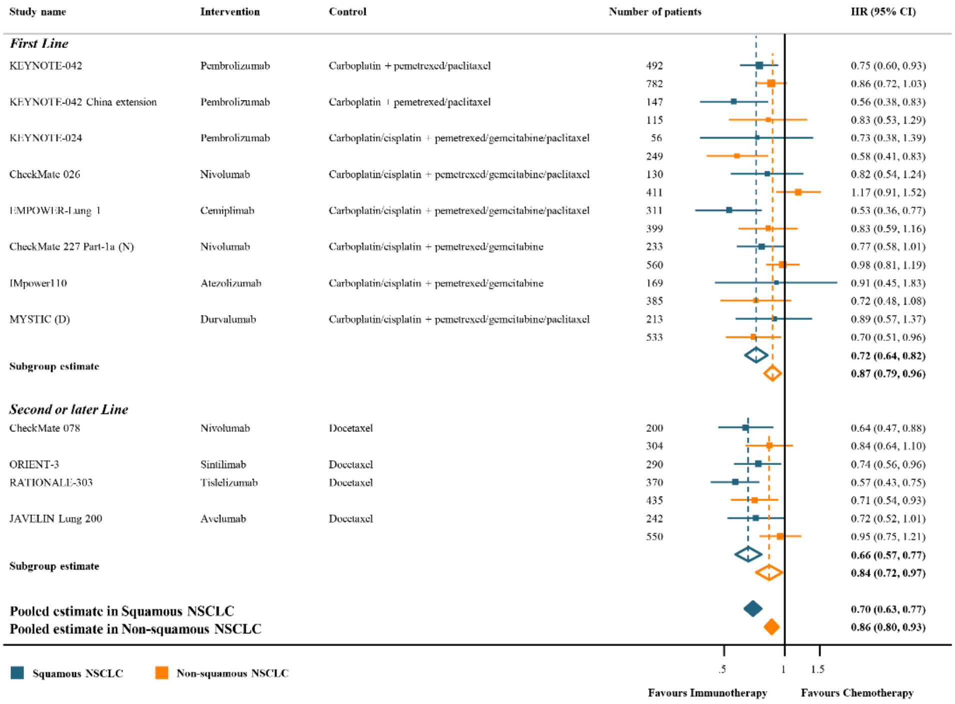

Supplementary Figure 5: Overall survival benefits of ICI monotherapy compared with chemotherapy according to histology in all included studies after sensitivity analysis. ICI, immune checkpoint inhibitor; HR, hazard ratio; RHR, ratios of the HRs; CI, confidence interval.

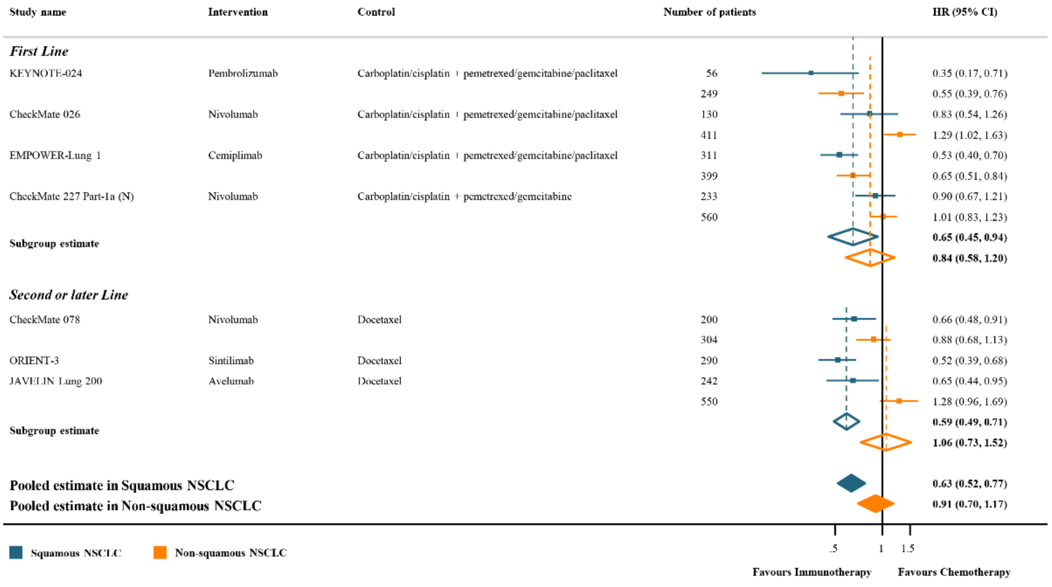

Supplementary Figure 6: Progression-free survival benefits of ICI monotherapy compared with chemotherapy according to histology in all included studies after sensitivity analysis. ICI, immune checkpoint inhibitor; HR, hazard ratio; RHR, ratios of the HRs; CI, confidence interval.

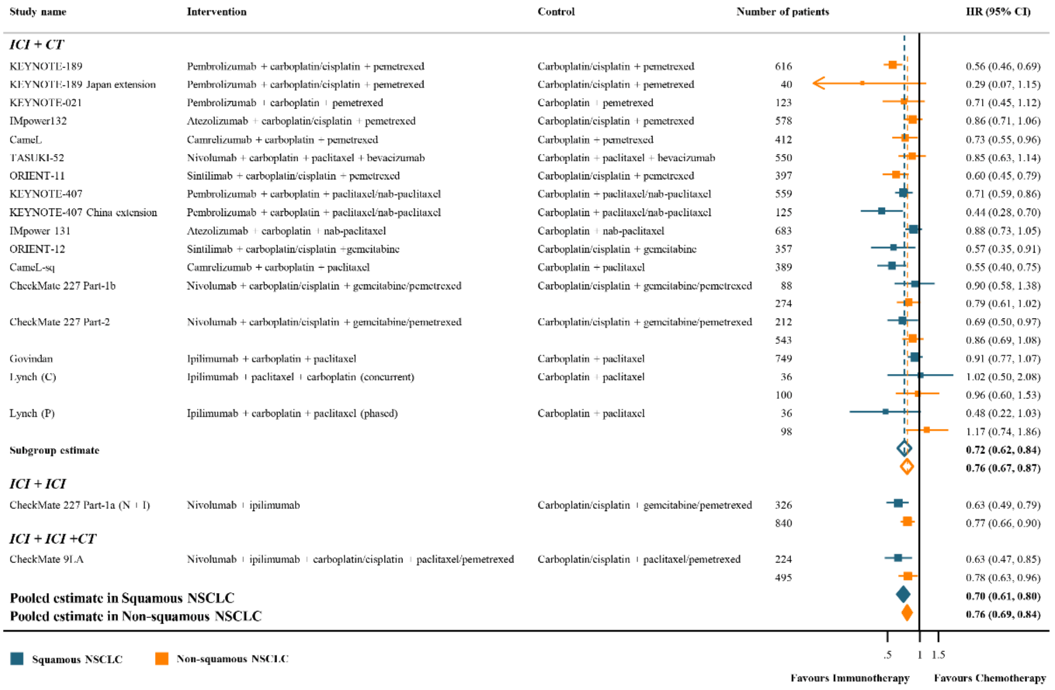

Supplementary Figure 7: Overall survival benefits of ICI-based combination treatments compared with chemotherapy according to histology in all included studies after sensitivity analysis. ICI, immune checkpoint inhibitor; HR, hazard ratio; RHR, ratios of the HRs; CI, confidence interval.

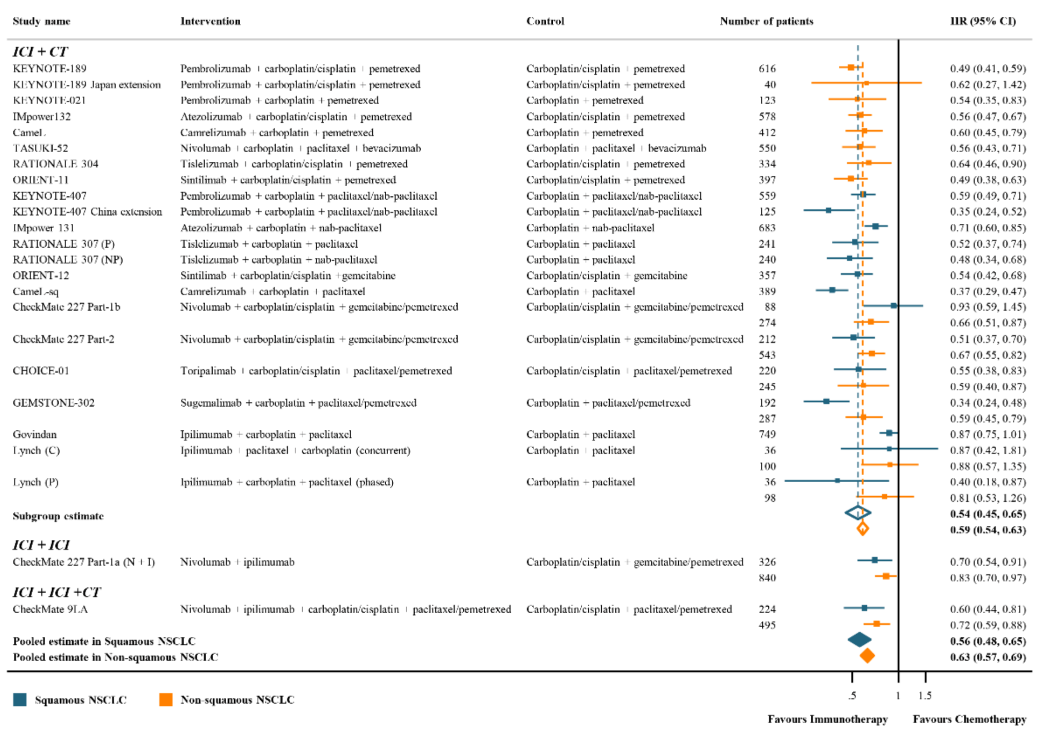

Supplementary Figure 8: Progression-free survival benefits of ICI-based combination treatments compared with chemotherapy according to histology in all included studies after sensitivity analysis. ICI, immune checkpoint inhibitor; HR, hazard ratio; RHR, ratios of the HRs; CI, confidence interval.

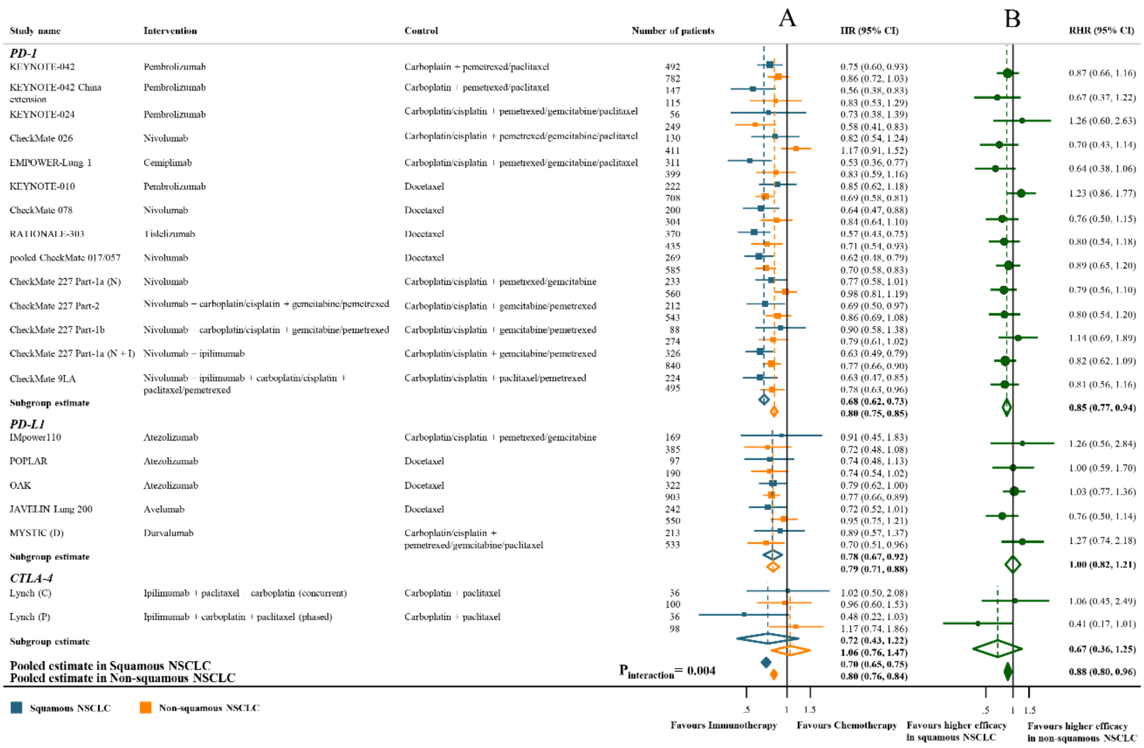

Supplementary Figure 9: Overall survival benefits of ICI compared with chemotherapy stratified by the class of ICI in studies with paired subgroups. (A) HRs according to histology. (B) Trial-specific ratios of the HR in SQ-NSCLC to the HR in non-SQ-NSCLC. ICI, immune checkpoint inhibitor; HR, hazard ratio; RHR, ratios of the HRs; CI, confidence interval.

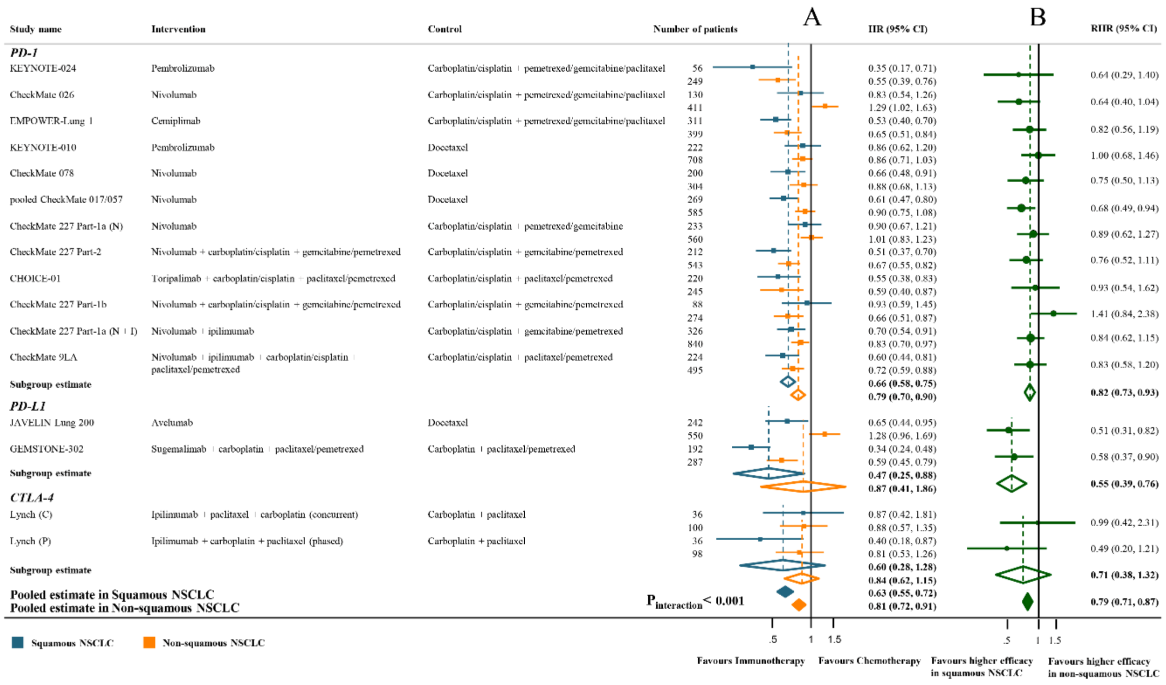

Supplementary Figure 10: Progression-free survival benefits of ICI compared with chemotherapy stratified by the class of ICI in studies with paired subgroups. (A) HRs according to histology. (B) Trial-specific ratios of the HR in SQ-NSCLC to the HR in non-SQ-NSCLC. ICI, immune checkpoint inhibitor; HR, hazard ratio; RHR, ratios of the HRs; CI, confidence interval.

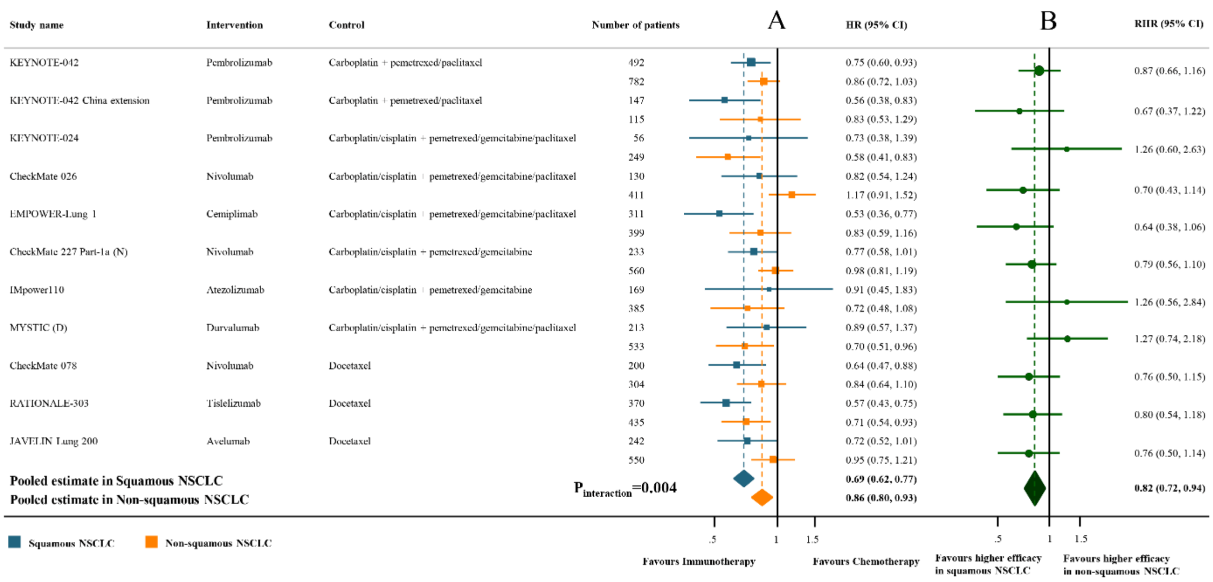

Supplementary Figure 11: Overall survival benefits of ICI monotherapy compared with chemotherapy in studies with paired subgroups after sensitivity analysis. (A) HRs according to histology. (B) Trial-specific ratios of the HR in SQ-NSCLC to the HR in non-SQ-NSCLC. ICI, immune checkpoint inhibitor; HR, hazard ratio; RHR, ratios of the HRs; CI, confidence interval.

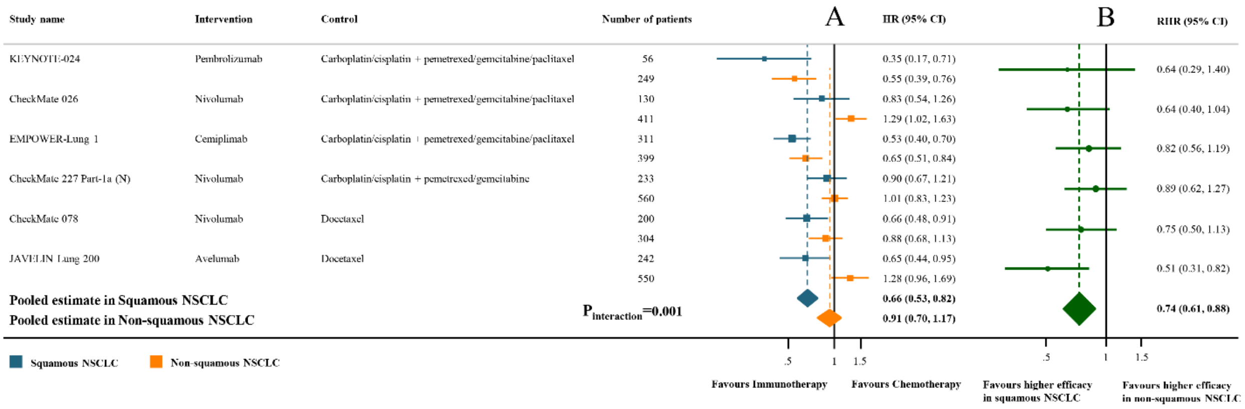

Supplementary Figure 12: Progression-free survival benefits of ICI monotherapy compared with chemotherapy in studies with paired subgroups after sensitivity analysis. (A) HRs according to histology. (B) Trial-specific ratios of the HR in SQ-NSCLC to the HR in non-SQ-NSCLC. ICI, immune checkpoint inhibitor; HR, hazard ratio; RHR, ratios of the HRs; CI, confidence interval.
